# Historic hybridization and introgression between two iconic Australian anemonefish and contemporary patterns of population connectivity

**DOI:** 10.1002/ece3.251

**Published:** 2012-07

**Authors:** M H van der Meer, G P Jones, J-P A Hobbs, L van Herwerden

**Affiliations:** 1 Molecular Ecology and Evolution Laboratory, Australian Tropical Sciences and Innovation Precinct, James Cook UniversityTownsville 4811, Australia; 2 School of Marine and Tropical Biology, James Cook UniversityTownsville 4811, Australia; 3 ARC Centre of Excellence for Coral Reef Studies, James Cook UniversityTownsville 4811, Australia; 4 The Oceans Institute and School of Plant Biology, The University of Western AustraliaCrawley 6009, Australia

**Keywords:** *Amphiprion*, coral reef fish, endemism, extinction risk, Great Barrier Reef, isolated islands, Lord Howe Island

## Abstract

Endemic species on islands are considered at risk of extinction for several reasons, including limited dispersal abilities, small population sizes, and low genetic diversity. We used mitochondrial DNA (D-Loop) and 17 microsatellite loci to explore the evolutionary relationship between an endemic anemonefish, *Amphiprion mccullochi* (restricted to isolated locations in subtropical eastern Australia) and its more widespread sister species, *A. akindynos*. A mitochondrial DNA (mtDNA) phylogram showed reciprocal monophyly was lacking for the two species, with two supported groups, each containing representatives of both species, but no shared haplotypes and up to 12 species, but not location-specific management units (MUs). Population genetic analyses suggested evolutionary connectivity among samples of each species (mtDNA), while ecological connectivity was only evident among populations of the endemic, *A. mccullochi*. This suggests higher dispersal between endemic anemonefish populations at both evolutionary and ecological timeframes, despite separation by hundreds of kilometers. The complex mtDNA structure results from historical hybridization and introgression in the evolutionary past of these species, validated by msat analyses (NEWHYBRIDS, STRUCTURE, and DAPC). Both species had high genetic diversities (mtDNA *h* > 0.90, π = 4.0%; msat genetic diversity, *gd* > 0.670). While high *gd* and connectivity reduce extinction risk, identifying and protecting populations implicated in generating reticulate structure among these species should be a conservation priority.

## Introduction

Remote islands often contain a disproportionate number of endemic species (Ceballos and Brown 1995; Randall 1998; Gillespie et al. 2008) and genetically distinct populations of species with broad geographic ranges (Avise 1992; Slatkin 1993). The uniqueness of island communities makes them a high conservation priority (Gillespie and Roderick 2002), even more so given the extremely high rates of local and global extinctions of species inhabiting islands (Steadman 1995). Island endemics face an elevated risk of extinction because they often have vulnerable traits, such as, limited dispersal abilities, small population sizes, and low genetic diversity (*gd*) due to genetic drift and inbreeding (Frankham 1997, 1998). A sound knowledge of the evolutionary history and adaptive capacity of endemic species or isolated populations is required in order to understand what increases their risk of extinction, thereby enabling the development of appropriate conservation measures (Faith 1992; Moritz 2002).

Throughout the world's tropical oceans, coral reef organisms are distributed on islands and reefs that represent varying degrees of isolation. For coral reef fish, it is the most isolated locations that contain the highest levels of endemism (Lessios et al. 2001; Jones et al. 2002; Allen 2008). Isolated islands also support genetically differentiated populations of some widespread reef fish species (Muss et al. 2001; Planes and Fauvelot 2002; Winters et al. 2010). Islands communities are also characterized by a high proportion of vagrants, which can result in a high incidence of hybridization due to a scarcity of conspecific mates (Hobbs et al. 2009b). Reef fish on isolated islands also appear to be more vulnerable to extinction as evidence by recent extinctions (Roberts and Hawkins 1999; Dulvy et al. 2003). These extinctions highlight the need to examine the genetic characteristics of island fish faunas to help determine why this group may be vulnerable. Processes that influence genetic resilience and promote species persistence times may be identified by examining spatial and temporal patterns of gene flow.

Coral reef anemonefish (genus *Amphiprion*) represent a useful model system for understanding evolutionary histories and population genetic structures of island fish faunas. Although the genus has a broad Indo–Pacific distribution, more than 25% of species are endemic to isolated islands or have peripheral populations at these remote locations (Fautin and Allen 1997). Although these island endemics are often closely related to more broadly distributed sister species, the historic colonization and speciation processes, and current levels of population differentiation are not well known. In a detailed phylogenetic study of 23 of the 28 anemonefish, Santini and Polacco (2006) suggested the group (Family: Pomacentridae, subfamily Amphiprioninae) originated some 5–13 million years ago in the Indo–Pacific, with many of the endemics being of recent origin. A more detailed investigation of their historic relationships with putative sister species, and current levels of gene flow among locations, is necessary to understand how endemic anemonefish originate and persist. This information will be useful for predicting how these species will persist in the future and aid management strategies aimed at conserving these iconic coral reef fish.

Using phylogenetic and population genetic analyses, we reconstructed the evolutionary history of two Australian anemonefish: a small-range species, *Amphiprion mccullochi* (endemic to Middleton Reef [MR], Elizabeth Reef [ER], and Lord Howe Island [LHI]) and its more widespread sister species, *A. akindynos,* found on the Great Barrier Reef, New Caledonia and the subtropical east coast of Australia. The endemic McCulloch's anemonefish is of particular conservation concern because it has three characteristics known to elevate the risk of extinction—a very small geographic range (Coleman 1980; Fautin and Allen 1992; Hobbs et al. 2009a), extreme habitat specialization (one species of host anemone—*Entacmaea quadricolor;*Fautin and Allen 1992) and relatively small local populations (Choat et al. 2006; Hobbs and Feary 2007). *Entacmaea quadricolor* is distributed from Micronesia and Melanesia to East Africa and the Red Sea and to Japan and Australia (Fautin and Allen 1992), but no further south than the Solitary islands (NSW) on the east coast of Australia. *Amphiprion mccullochi* is thought to have arisen by divergence from a more widespread most recent common ancestor (mrca) shared with *A. akindynos*, its sister species, on the eastern Australian coast (Santini and Polacco 2006). However, the mode of speciation and current levels of genetic connectivity among the three populations of *A. mccullochi* are unknown. *Amphiprion akindynos* may be less of a conservation concern because it is more widely distributed, inhabits six species of anemones (Fautin and Allen 1997), and can be locally abundant (e.g., Richardson 1999).

This study addressed the following specific questions: (1) What is the evolutionary relationship between the sister species, *A. mccullochi* and *A akindynos*, based on mitochondrial DNA (mtDNA)? (2) How many management units (MUs, sensu Moritz 1994) can be identified for *A. mccullochi* and *A. akindynos*? (3) What is the contemporary relationship of connectivity between and within species, based on msat DNA? (4) What are the genetic diversities of these sister species and do they suggest resilience or susceptibility to extinction and environmental change? Identifying MUs, including the direction of connectivity between these units, is essential information to ascertain best practice management and maximize biodiversity conservation of these important southernmost coral reefs. We discuss the implications of the underlying genetic structure to extinction risk and the conservation of these remote populations.

## Materials and Methods

### Study system

McCulloch's anemonefish (*A. mccullochi*) has the smallest geographic range of any of the 28 species of anemonefish and is endemic to three isolated, oceanic locations more than 600 km off the east Australian coastline (ER, MR, and LHI). It is only found living in close association with its host anemone, *E. quadricolor,* and occurs at depths between 2 and 45 m (Fautin and Allen 1992). Its coloration of black body with a whitish snout, caudal peduncle, and caudal fin makes it easily recognizable. Its sister species, *A. akindynos* (Santini and Polacco 2006) is more widespread, ranging from the Great Barrier Reef (GBR) south to the Solitary Islands and extending out to New Caledonia (but not including ER and MR or LHI). It lives among its host anemones *E. quadricolor*, *Heteractis aurora*, *H. crispa*, *H. magnifica*, *Stichodactyla haddoni*, and *S. mertensii* (Fautin and Allen 1992), at depths between 1 and 25 m (Allen 1991). It has two white bars on its body and a color transition from a dark brown/orange body to a whitish caudal fin (Fautin and Allen 1992).

### Sampling locations and procedures

Finclips from 60 *A. mccullochi* individuals were collected from two out of the three known populations at MR (*n* = 30; Choat et al. 2006) and from LHI (*n* = 30; Hobbs et al. 2009a), and preserved in 70% alcohol. These two populations (MR and LHI) represent either end of the entire geographic range of this species, spanning 200 km. Bay et al. (2006) sampled *A. akindynos* at two GBR locations—a central, Lizard Island (LI) population (*n* = 20) and a southern peripheral, One Tree Island (OTI) population (*n* = 24), spanning 1200 km. The geographic ranges of the two sister species are separated by at least 600 km of deep open ocean habitat.

### Genetic techniques

Standard DNA laboratory and analytical procedures were used for mtDNA sequencing (Bay et al. 2006, van Herwerden et al. 2009). Total genomic DNA was extracted from approximately 1 mm^3^ of tissue using standard chelex-proteinase K digestion extraction procedures (Walsh et al. 1991). Each 20-μL polymerase chain reaction (PCR) amplification reaction contained 20 ng DNA, 0.2 mM DNTP, 1 unit of Bioline Biotaq Red DNA Polymerase, 0.5 µM of each primer, and variable MgCl_2_. The noncoding regions of the mtDNA were selectively amplified by polymerase chain reaction (PCR) using the following primers for the damselfish *Acanthachromis polyacanthus*: dLoop F (5′-CATATATGTRTTATCAACATTA-3′) and CR-E H16498R (5′-CCTGAAGTAGGAACCAGATG-3′) (Bay et al. 2006). Primers were tested and optimized using a Bio-Rad C1000 Thermal Cycler (Bio-Rad, Australia). Amplifications followed the same basic cycling protocol: 40 sec at 94°C, 40 sec at primer-specific annealing temperatures (two different touchdown profiles of five cycles at 55°C followed by 30 cycles at 53°C or five cycles at 53°C followed by 30 cycles at 51°C) and 40 sec at 72°C. The cycling profile was flanked by an initial 3-min denaturing step (94°C) and a 5-min terminal extension phase (72°C). Furthermore, we tested and sequenced a 16S mitochondrial marker (Santini and Polacco 2006) on 10 individuals per species (*A. mccullochi* and *A. akindynos*). The resulting phylogeographic tree was poorly resolved and provided no resolution. Thus, only D-loop results are presented in this study to identify haplotypic differences. We also genotyped both *A. mccullochi* and *A. akindynos* using 17 microsatellite markers developed for *A. mccullochi* (van der Meer et al. 2011). Six multiplex reactions were performed and each 10-μL PCR amplification reaction contained 10 ng DNA, 5 μL (2×) Type-it Multiplex PCR Master Mix, 2 μL multiplex primer mix (at a concentration of 2 µM). Amplifications followed the same basic cycling protocol: 30 sec at 95°C, 90 sec at 58°C, and 40 sec at 72°C. The cycling profile was flanked by an initial 5-min denaturing step (94°C) and a 30-min terminal extension phase (72°C). PCR products were purified by isopropanol precipitation for direct sequencing of D-loop (Macrogen, Korea), and ethanol ammonium acetate precipitation for genotyping microsatellite reactions (Genetic Analysis Facility, James Cook University, Townsville).

### mtDNA analysis

Forward sequences were automatically aligned using the plugin CLUSTAL W in Geneious Pro 4.7 (Drummond et al. 2009), conservatively trimmed to minimize the amount of missing data, and manually edited, inserting gaps where required and checking for ambiguities. Differences between individual sequences were determined for the following characters: A, G, T, C, and IUB symbols (Nomenclature Committee 1985). Sequence data were obtained from GenBank for the following five species that acted as outgroups: *A. clarkii* (DQ343928.1), *A. clarkii* (orange; DQ343929.1), *A. chrysopterus* (DQ343927.1), *A. latezonatus* (DQ343933.1), and *A. leucokranos* (DQ343934.1). All *A. akindynos* sequences (DQ250449.1 to DQ250492.1) from Bay et al. (2006) were analyzed together with *A. mccullochi* sequences based on the close genetic relationship between these two species (Santini and Polacco 2006). One GBR *A. akindynos* sequence from an unspecified location was also included (DQ343924.1).

jModeltest (Posada 2008) identified an HKY + I model under Akaike Information Criterion with gamma = 0.271. Fifty-three of the 317 nucleotides sequenced for *A. mccullochi* were parsimony informative. The transition (ts): transversion (tv) substitution ratio was approximately 6:1. There were a total of 65 variable sites excluding five single base indels. The nucleotide composition was AT biased with 71.11% AT:28.89% GC (*A. mccullochi* Dloop), 71.60% AT:28.40% GC (*A. akindynos* Dloop), and 71.36% AT:28.64% GC (combined *A. mccullochi* and *A. akindynos* Dloop), which is consistent with fish mitochondrial DNA (McMillan and Palumbi 1997).

**Figure 1:**
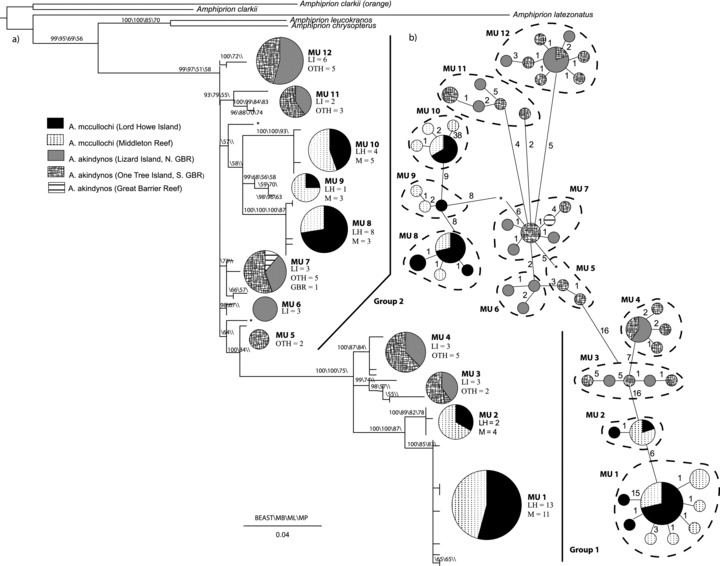
(A) An outgroup rooted phylogram of D loop sequences from 50 *Amphiprion mccullochi* individuals from Middleton Reef and Lord Howe Island and 44 *A. akindynos* individuals from Lizard and One Tree Islands on the Great Barrier Reef. *Amphiprion clarkii* was specified as the outgroup species. This represents the best ML tree from 10 individual runs in GARLI. Asterisks identify individuals of *A. akindynos* that fall outside a clade. Numbers on branches indicate support for each clade, BEAST, MB, ML, and MP. (B) Haplotype minimum spanning tree (MST) with number of substitutions between haplotypes indicated on connectors. Different shading represents each of the four locations as shown on the key to the figure.

The data underwent four phylogenetic analyses (Fig. 1A): (1) Bayesian inference (MB) in MrBayes 3.1 (Huelsenbeck et al. 2001, 2002) with 10 million generations of Monte Carlo Markov chains (MCMC); (2) ten independent maximum likelihood (ML) analyses, followed by an 100 bootstrap replicate analysis using GARLI 0.951 (Zwickl 2006), from which a 50% majority rule ML consensus tree was constructed in PAUP* 4.10b (Swofford 2001); (3) maximum parsimony (MP) was performed in MEGA 4.0 (Tamura et al. 2007) with 1000 bootstrap replicates, from which a 50% majority rule consensus tree was constructed and; (4) Bayesian inference in BEAST V1.6.1 (Drummond and Rambaut 2007) was tested using a strict clock model (estimated clock rate, uniform prior distribution, HKY+G site model for 5 million MCMC chains with sampling at every 5000 trees) and a relaxed clock model (uncorrelated lognormal clock model, with the same parameters noted previously). Tracer V1.5 identified no significant difference between clock models based on the Bayes Factor (BF) evaluation of the models (Kass and Raftery 1995; Suchard et al. 2001), BF = 0. We therefore used the speciation (Yule process) to construct a Bayesian skyline plot using a strict clock model (as above, but with 50 million MCMC chains sampled every 5000th tree). Maximum clade credibility trees (MCCT) were constructed in TreeAnnotator V1.6.1 after discarding the initial burn-in of 10%. The MCCT was viewed separately in FigTree V1.3.1 (available at http://tree.bio.ed.ac.uk/software/figtree/). The best outgroup rooted ML tree from GARLI was selected to reconstruct the evolutionary history with bootstrap values for each clade from all four analyses, if present (Fig. 1A). A minimum spanning tree (MST) of the mtDNA sequence data was computed using Arlequin ver. 3.5 (Excoffier et al. 2005) to identify shared haplotypes between locations sampled and/or species (Fig. 1B).

Bayesian skyline plots in BEAST V1.6.1 were constructed to evaluate the presence of demographic stability and/or expansions from coalescent analyses using strict clock model (parameters as above). We also evaluated population stasis using Fu's *F*s parameter for population stasis (Fu 1997) and Tajima's D test for selective neutrality of mtDNA (Tajima 1983), both of which accepted the hypothesis of a static population under an assumption of selective neutrality for *A. mccullochi* (D = 1.578, *P* = 0.96 and *F*s = 2.80, *P* = 0.85); however, for *A. akindynos* selective neutrality of the mtDNA was accepted (D = 0.103, *P* = 0.61), while population stasis was rejected (*F*s = –24.42, *P* < 0.0001), suggesting a spatial expansion by *A. akindynos.* Following 90,000 re-samplings of the data, the *F*-statistics (fixation indices, Φ*_st_*, Φ*_ct_*, Φ*_sc_*) were determined using an analysis of molecular variance (AMOVA) (Excoffier et al. 1992) to detect population genetic partitioning between either regions (LI and OTI vs. MR and LHI) or genetically distinct lineages regardless of location or species (MUs were compared to each other; Table 1). Haplotype (*h*) and nucleotide diversities (%π) of the data were interpreted based on Grant and Bowen (1998). Population pairwise *F*_st_ comparisons (measured in Arlequin ver. 3.5) initially identified no differences between locations for either species, which informed further inter- and intraspecific analyses between clades (Table 2). Isolation by distance (IBD) between reefs was tested using a Mantel test in IBD ver1.4 with 10,000 permutations (Bohonak 2002).

**Table 1:** Analysis of molecular variance (AMOVA) analysis for the genetic data from *Amphiprion mccullochi* and *A. akindynos* structured into (A) mtDNA—partitioned by geographic region (GBR vs. offshore locations) and (B) mtDNA—partitioned by ESUs, and (C) msat DNA—partitioned by geographic region (GBR vs. offshore locations)

Source of variation	df	SSD	Variance component	Percentage of variation	*F*-statistics fixation indices (*P*-value)
(A) Region					
Among groups	1	170.34	3.53	32.63	*F*_ct_ = 0.326
					(0.341 ± 0.014)
Among populations within groups	2	10.10	−0.10	−0.93	*F*_sc_ = –0.014
					(0.596 ± 0.011)
Within populations	90	665.51	7.39	68.31	*F*_st_ = 0.317
					**(<0.001 ± 0.000)**

(B) Clades					
Among groups	1	352.29	6.38	49.56	*F*_ct_ = 0.496
					**(0.007 ± 0.003)**
Among populations within groups	10	367.64	5.13	39.88	*F*_sc_ = 0.791
					**(<0.001 ± 0.000)**
Within populations	80	108.74	1.36	10.56	*F*_st_ = 0.894
					**(<0.001 ± 0.000)**

(C) Microsatellite					
Among groups	1	30.54	0.25	5.01	*F*_ct_ = 0.05
					(0.337 ± 0.002)
Among populations within groups	2	13.684	0.04	0.85	*F*_sc_ = 0.009
					(0.006 ± 0.000)
Within populations	192	917.64	4.78	94.14	*F*_st_ = 0.05
					**(<0.001 ± 0.000)**

**Table 2:** Genetic diversity estimates for *Amphiprion mccullochi* and *A. akindynos*. Sample size (*n*), number of haplotypes (*n*_h_), haplotypes diversity ± SE (*h*), nucleotide diversity ± SE (%π), and genetic diversity (*gd*). Genetic diversity estimates for one *A. akindynos* sample of unknown location on the GBR was omitted

Site	*n*	*n*_h_	*h*	%π	*gd*
*A. mccullochi*					
Middleton Reef	22	14	0.952 ± 0.026	4.90 ± 2.53	0.691 ±0.360
Lord Howe Island	26	11	0.846 ± 0.054	5.11 ± 2.62	0.670 ± 0.351
**Total**	48	21	0.905 ± 0.027	5.00 ± 2.50	0.690 ± 0.370
Clade 1.1	22	9	0.701 ± 0.103	1.76 ± 0.98	
Clade 1.2	6	3	0.600 ± 0.215	0.11 ± 0.14	
Clade 2.4	11	4	0.600 ± 0.154	0.22 ± 0.20	
Clade 2.5	3	3	1.000 ± 0.272	0.63 ± 0.60	
Clade 2.6	8	4	0.643 ± 0.184	0.24 ± 0.22	

*A. akindynos*					
Lizard Island	20	14	0.952 ± 0.026	4.90 ± 2.53	0.718 ± 0.371
One Tree Island	24	11	0.846 ± 0.054	5.11 ± 2.62	0.693 ± 0.360
**Total**	44	34	0.976 ± 0.013	4.03 ± 2.06	0.706 ± 0.484
Clade 1.3	5	5	1.000 ± 0.127	1.46 ± 1.00	
Clade 1.4	8	4	0.643 ± 0.184	0.40 ± 0.32	
Clade 2.1	2	2	1.000 ± 0.500	0.32 ± 0.45	
Clade 2.2	3	3	1.000 ± 0.272	0.63 ± 0.60	
Clade 2.3	8	6	0.893 ± 0.111	0.76 ± 0.52	
Clade 2.7	5	4	0.900 ± 0.161	1.20 ± 0.85	
Clade 2.8	11	7	0.818 ± 0.119	0.56 ± 0.40	

*A. mccullochi* and *A. akindynos*					
**Total**	93	55	0.969 ± 0.009	5.67 ± 2.81	0.691 ± 0.370

### Microsatellite analyses

GENEPOP 4.0 (Rousset 2008) was used to perform exact tests of departures from Hardy–Weinberg equilibrium (HWE) for each locus per sampled location (i.e. 17 loci × four sampled locations) and to test for linkage disequilibrium (LD) between the 17 loci within each of the two study species (i.e., 17 × 17 (–17) = 272 tests for each species), using the Markov chain algorithm. If departure from HWE was observed, the program MICRO-CHECKER 2.2.3 (van Oosterhout et al. 2004) was used to detect the presence of null alleles, large allele dropout, and other scoring errors. We conducted 20 batches with 5000 iterations per batch. A false discovery rate (FDR; Benjamini and Hochberg 1995) correction was applied to all HWE and LD results, using the program QVALUE (Storey 2002). Significant single-locus departures from HWE were detected in eight of sixty-eight tests at the population level before FDR correction and five afterwards (Am1, Am11, Am14, Am17, Am19). Null alleles were identified in LI (Am11, Am17, Am19), OTI (Am1, Am7, Am21), MR (Am11, Am14, Am17), and LHI (Am11, Am17, Am19) as indicated in MICROCHECKER. Loci that were not in HWE and had null alleles (i.e., Am1, Am11, Am14, Am17, Am19) were not used in subsequent analysis (ARLEQUIN, STRUCTURE, NEWHYBRIDS, and MIGRATE-n). Of 544 locus × locus exact tests of LD, (272 per species), only 13 were significant before FDR and none after FDR correction (Benjamini and Hochberg 1995), indicating that loci are independently assorted.

Following 90,000 resamplings of the data, *F*-statistics (fixation indices, Φ_st_, Φ_ct_, Φ_sc_) were determined using an AMOVA (Excoffier et al. 1992) to detect population genetic partitioning between regions (LI and OTI vs. MR and LHI; Table 1). Microsatellite *gd* of differentiated populations was determined in ARLEQUIN 3.5 (Excoffier et al. 2005), using 90,000 permutations (Table 2). IBD was tested using a Mantel Test in IBD ver1.4 with 10,000 permutations (Bohonak 2002), as noted for mtDNA analyses.

We used STRUCTURE V2.3 (Pritchard et al. 2000; Hubisz et al. 2009) to identify groups of randomly mating individuals based on microsatellite allele frequencies. Data were first tested using the “admixture” and “no admixture” models, including information about the sampling location as a prior and correlated allele frequencies. Two repetitions were run for each possible K value, informed by the number of partitions identified by mtDNA analyses, so K ranged between 1 and 12. Short 20,000 MCMC iterations were performed per analysis and a 1000 tree burn-in was applied. To determine the “best value” for K, we followed the method suggested by Pritchard et al. (2000), which involved comparing mean log likelihoods penalized by one-half of their variances (see Hubisz et al. 2009). We also chose an “admixture” model including information about the sampling location as a prior and correlated allele frequencies, with two repetitions of K (K = 2), for 1 million MCMC iterations and a 10,000 tree burn-in, as this was the best K value identified in the initial analyses.

Given contrasting results found with mtDNA and msat DNA, we specifically tested for interbreeding using two programs: (1) NEWHYBRIDS (Anderson and Thompson 2002), which implements a Bayesian method aimed at detecting the presence of hybrids from a sample of individuals of mixed origin. We used a MCMC procedure with a 150,000 burn-in and 150,000 steps, and (2) MIGRATE-n 2.4.3 (http://popgen.sc.fsu.edu/Migrate-n.html; see Beerli and Felsenstein 2001; Beerli 2004) to estimate long-term gene flow between MUs and short-term gene flow (nuclear DNA) between locations. We set the migration rate parameter for mtDNA (Theta and M to a maximum of 0.1 and 5000, respectively) and msatDNA (both Theta and M to a maximum of 100). We conducted 10 replicates of a Bayesian analysis with one long-chain sampling every 100 trees of 100,000 sampled and a 20,000 iteration burn-in for mtDNA and a Bayesian analysis with one long-chain sampling every 100 trees of 5000 sampled and a 1000 iteration burn-in for msatDNA. All parameters converged and fell within the 90% CI, yielding values for θ and *m* for each locus per population. Finally, a discriminant analysis of principal components (DAPC) was also used to determine contemporary gene flow between populations as per Jombart et al. (2010). This required the use of the program R 2.12 (http://www.Rproject.org). We retained 75 principal components (PCs) comprising 95% of the total genetic information as predictors for DA.

## Results

### Evolutionary relationship

The best outgroup rooted ML phylogram and the MST identified two strongly supported evolutionary groups and this was supported by all additional phylogenetic analyses (Fig. 1A). It appeared that either one evolutionary group emerged from the other or that there was a splitting to form two sister groups, depending on the analysis. The two evolutionary groups did not represent the two species per se, and lacked reciprocal monophyly, because individuals of both species were detected in both evolutionary groups. Despite the lack of species-specific partitions, there were no shared haplotypes between species. This suggests a complex evolutionary history between these recognized species or incomplete lineage sorting. Sixteen synapomorphic substitutions characterized the split between these two phylogenetic groups (1 and 2). Group 1, which emerges from Group 2, contains 56% of all sampled *A. mccullochi,* whereas the apparently older Group 2 contains 70% of all sampled *A. akindynos* represented.

### Management units

The two evolutionary groups contained a total of seven and possibly as many as 12 MUs, of which three to four were in Group 1 (MU1–4) and four to eight (MU5–12) were in Group 2. Haplotype sharing was observed between locations within species: MR—LHI in all five *A. mccullochi* MUs; LI—OTI in five of the seven *A. akindynos* MUs. When samples were structured by MU irrespective of sampling location (MU1–12) during population genetic analyses, pairwise *F*_st_ values indicating significant genetic differentiation were obtained between all but two MU pairs (pairwise *F*_st_ = 0.455–0.983, *P* < 0.00001 to 0.04800). Exceptions were MU5–6 (pairwise *F*_st_ = 0.634, *P* = 0.10) and MU5–9 (*F*_st_ = 0.860, *P* = 0.10). Lack of significance among these MU-pairs, despite very large pairwise *F*_st_ values may result from smaller sample sizes in these three clades than in the remaining nine clades (see Fig. 1A). AMOVA results confirmed genetic partitioning between MUs, Φ_st_ = 0.894 (*P* < 0.001); however, this only explained 10.56% of the variation, while 49.56% and 39.88% of the variation occurred among groups (1 vs. 2) and among populations within groups, respectively (Φ_ct_ = 0.496, *P* < 0.001; Φ_sc_ = 0.791, *P* < 0.001; Table 1).

We further examined levels of gene flow between locations and/or species using pairwise *F*_st_ values for a number of interspecies and mixed-species comparisons, consistent with the phylogenetic signals described above. There was no mtDNA differentiation between sample locations (LHI vs. MR or OTI vs. LI) within either species (*A. mccullochi* or *A. akindynos*, *F*_st_ = –0.005 and –0,025, *P* = 0.414 and 0.865, respectively). Given this and disregarding species identity, to compare fish from offshore to fish from continental shelf locations (OTI or LI vs. LHI or MR), all combinations of comparisons showed significant pairwise mtDNA *F*_st_ values (*F*_st_ = 0.293–0.349, *P* < 0.00001). The more statistically rigorous AMOVA of samples structured by region (LHI and MR vs. OTI and LI) confirmed mtDNA genetic partitioning with more than two-thirds (68.31%) of the genetic variation within locations, Φ_st_ = 0.317 (*P* < 0.0001), and less than one-third of the variation detected among regions (offshore vs. GBR continental locations), which was not significant, Φ_ct_ = 0.327 (*P* = 0.341). Further, none of the variation occurred among locations within regions (i.e., LHI vs. MR; OTI vs. LI), Φ_sc_ = –0.014 (*P* = 0.596). There was no IBD based on mtDNA from all locations sampled, using a Mantel test of pairwise geographic (km) and genetic (*F*_st_) distance between locations (*z* = 2079.08, *R*^2^ = 0.31, *P* = 0.337).

Both Fu's *F*s parameter for population stasis (Fu 1997) and Tajima's D test for selective neutrality of mtDNA (Tajima 1983) accepted the hypothesis of a static population under an assumption of selective neutrality for *A. mccullochi* (D = 1.578, *P* = 0.96 and *F*s = 2.80, *P* = 0.85). In contrast, population stasis was rejected for *A. akindynos* (D = –24.42, *P* < 0.0001; *F*s = –11.757, *P* = 0.02), suggesting that this species has undergone spatial expansion. This was confirmed by both Bayesian and mismatch distribution analyses (data not shown). When mtDNA data of specimens from all locations, regardless of species partition, were considered collectively (MR vs. LHI vs. OTI vs. LI), total mtDNA suggested selective neutrality (D = 0.103–0.636, *P* = 0.61–0.80) and appeared to be either evolutionarily stable or to represent admixture of previously differentiated lineages.

### Contemporary connectivity

Msat pairwise *F*_st_ values were largely consistent with mtDNA results: There was no genetic differentiation between *A. mccullochi* populations, LHI-MR (*F*_st_ = 0.005, *P* = 0.081), but significant differentiation existed between *A. akindynos* populations, LI and OTI (*F*_st_ = 0.016, *P* = 0.036). Importantly, the more statistically rigorous AMOVA of samples structured by region (LHI and MR vs. OTI and LI) identified msat genetic partitioning within locations, accounting for 94.14% of the genetic variation; Φ_st_ = 0.06 (*P* < 0.001). Only 5.01% of the variation occurred among regions (offshore vs. GBR continental locations), but this was not significant, Φ_ct_ = 0.05 (*P* = 0.337), and almost none (0.85%) of the variation, albeit significant, occurred among locations within regions (i.e., LHI vs. MR, OTI vs. LI), Φ_sc_ = 0.009 (*P* = 0.006; Table 1). In contrast to mtDNA, there was strong evidence of IBD based on msat DNA using a Mantel test of log pairwise geographic (km) and log genetic (*F*_st_) distance between all sampled locations, regardless of species (*z* = –23.11, *R*^2^ = 0.89, *P* = 0.048).

**Figure 2:**
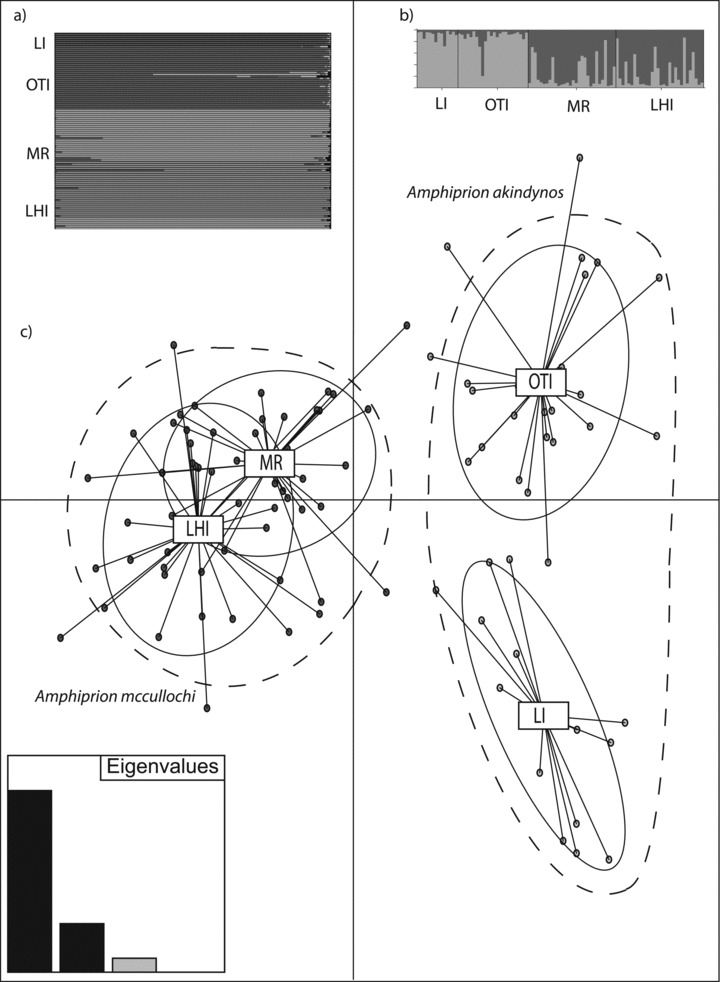
Separation of *Amphiprion mccullochi* and *A. akindynos* based on various analyses of msat loci: (A) NEWHYBRID analysis showing pure (gray) and F1 hybrid (black) status, (B) STRUCTURE cluster analysis, and (C) Scatterplots of the discriminant analysis of principal components (DAPC) of msat data for two *A. mccullochi* and two *A. akindynos* populations using geographic sample site as priors for genetic clusters. Populations are named and individual genotypes appear as dots surrounded by 95% inertia ellipses. Eigenvalues show the amount of genetic information contained in each successive principal component with *X* and *Y* axes constituting the first two principal components, respectively.

NEWHYBRIDS and STRUCTURE analyses indicated intraspecific but not interspecific gene flow between species and the likelihood of the marginal posterior probability distribution was greatest when *K* = 2 (Fig. 2A and B). DAPC was consistent with this and *A. mccullochi* populations (MR and LHI) appeared to have overlapping genotypic profiles, as per mtDNA, while *A. akindynos* populations (OTI and LI on the GBR) were genetically distinct from each other (Fig. 2C), unlike mtDNA results. Using the four sampled populations as a priori population criteria, DAPC assigned 96.94% of all individuals to the population where they were sampled (assignment per population, MR = 80%, LHI = 80%, LI = 100%, and OTI = 100%). MIGRATE-n indicated high levels of historical gene flow relative to contemporary gene flow. Levels of historical gene flow between lineages (i.e., Group 1 vs. Group 2; 4 Nm values ranged from 28 to 62) were an order of magnitude lower compared to within lineage geneflow: Group 1 (MU1.1–1.4; 4 Nm values ranged from 980 to 2326) and Group 2 (MU2.1–2.8; 4 Nm values ranged from 241 to 1247). Contemporary gene flow was a few orders of magnitude less than historical gene flow, with 4 Nm values ranging from 1 to 6.

### Genetic diversity

Genetic diversity was high for both species, evident from high haplotype (*h*), nucleotide (%π), and genotypic (*gd*) diversities for each sampled location (*h* = 0.846–0.982, %π = 3.97–5.11, *gd* = 0.670–0.718, for each species). Total haplotype, nucleotide, and genotypic diversity were also high when species were combined (*h* = 0.905–0.976, %π = 4.03–5.67, *gd* = 0.690–0.706; Table 2). When samples were grouped according to mtDNA evolutionary groups, irrespective of species or location, haplotype diversity was still high (*h* = 0.600–1.000), but nucleotide diversity was at least one-third (%π = 0.11–1.76) that of the aforementioned values for each species (Table 2).

## Discussion

Phylogenetic analysis of mtDNA revealed a complex evolutionary history where *A. mccullochi* and the more widespread *A. akindynos* were not partitioned into monophyletic lineages as anticipated, but were mixed into two paraphyletic lineages that did not correspond to either species or geographic location per se. These analyses also revealed that the species did not share any haplotypes with each other and identified up to 12 species-specific MUs distributed among the four geographic locations sampled for the two species. Population genetic analyses based on mtDNA confirmed the absence of genetic partitioning by location for both species examined. Importantly, despite this complex evolutionary history, msat DNA analyses revealed no contemporary gene flow existed between species, and geneflow between populations was limited. Populations connected by such low levels of gene flow are effectively demographically independent and significant to conservation as these MUs are fundamental to effective demographic management and are the logical unit for demographic study and population monitoring (Moritz 1994). Finally, the high genetic diversity of both species may result in greater adaptive capacity to cope with future environmental change, insofar as greater genetic diversity provides more “raw material” for selection to act on.

### Evolutionary relationship

There are three possible interpretations of the combined genetic results. First, the two species represent different color variants of a single species rather than different species per se. However, this contradicts existing taxonomic classification (Santini and Polacco 2006) and requires further ecological and experimental data to explore this. The second possibility is that incomplete lineage sorting may be responsible for the apparent lack of reciprocal mtDNA monophyly. However, as mtDNA lineages suffer incomplete lineage sorting for a much shorter period of time (25%) than do nuclear DNA lineages, our msat DNA results do not support this scenario, because msat DNA did partition the species, which leads to the third possible interpretation—that there has been historical bi-directional hybridization.

We argue that the high level of mtDNA genetic partitioning observed—while not identifying species-specific lineages or location-specific MUs—is most consistent with an evolutionary history of at least two reticulate events between *A. akindynos* and *A. mccullochi*. Reticulate events have been documented for numerous other reef fish species (e.g., McMillan et al. 1999; van Herwerden and Doherty 2006; van Herwerden et al. 2006; Yaakub et al. 2006; Marie et al. 2007), including secondary contact between differentiated lineages of *A. akindynos* on range edges as documented by Bay and Caley (2011). Hybridization and reticulate evolution is also common in other coral reef organisms (e.g., corals: Willis et al. 2006). Several lines of evidence support a scenario of historical hybridization: (1) two paraphyletic mtDNA lineages exist, each comprising of both *A. mccullochi* and *A. akindynos*, where each lineage may represent a mtDNA lineage of one of the species prior to historic hybridization; (2) there are no shared mtDNA haplotypes between present day populations of the two species; demographic geneflow is very limited between the species, as measured by msat DNA and Migrate-n; (3) there is population genetic partitioning between the two species, evident from both mtDNA and msatDNA, even if treated as populations rather than species and; (4) msat genotypes of almost 97% of individuals were assigned back to the population they were collected from and there was strong and total partitioning between species following DAPC analysis of msat DNA. Together, this suggests that geneflow between species occurred in the evolutionary past but is either no longer occurring, or is happening at a level not detectable in this study.

Historical gene flow between species via hybridization is increasingly being documented among coral reef fish (e.g., Yaakub et al. 2006; Hobbs et al. 2009b, in press) and has been reported in a number of anemonefish species, including *A. akindynos* (Fautin and Allen 1997; Sea Read 2009). Timm et al. (2008) reports hybridization between *Amphiprion* species, as a possible explanation for sequence sharing, especially since, within the genus *Amphiprion*, several species have similar coloration and overlapping variation at otherwise diagnostic morphological characters. Coloration is very pertinent in the present case, as juvenile *A. akindynos* and *A. mccullochi* are almost morphologically indistinguishable (Richardson 1998). Settlement to an anemone occurs during the juvenile phase and given that both species use the same host anemone (*E. quadricolor*; Fautin and Allen 1992) and have similar coloration, this could lead to the formation of heterospecific social groups, and possible interbreeding. Furthermore, although the two species occur in allopatry, two vagrant individuals of *A. akindynos* have been recorded at LHI (Crean et al. 2009). The arrival of vagrants into the distributional range of an allopatric sister species is thought to promote interbreeding between species due to the low availability of conspecific partners for the vagrants (Hobbs et al. 2009b). The East Australian Current flows through the ranges of the two study species and the strength and direction is influenced by climate (Philander et al. 1990; Middleton et al. 2009). Historical changes in this current may have facilitated the arrival of vagrants, which may have resulted in contact and hybridization between the two species. Taken together, all these factors suggest hybridization is a likely scenario in the evolutionary history of these two species.

### Management units

Two groups (evolutionary units) and at least seven MUs were identified in this study, with at least two *A. akindynos* MUs and five *A. mccullochi* MUs. While each MU represents one of the study species exclusively, most are not partitioned by geography. This complex underlying phylogenetic structure may have occurred because anemonefish have both self-recruitment at demographic scales (Jones et al. 2005) and interpopulation connectivity at longer evolutionary timescales (Timm et al. 2008).

### Contemporary connectivity

Microsatellite loci detected very limited geneflow between the sister species *A. akindynos* and *A. mccullochi*, which strengthens our suggestion of evolutionary hybridization as current day populations are not mixing. Likewise, LI and OTI *A. akindynos* populations were genetically distinct which is consistent with Bay and Caley (2011), indicating that the distance between these two populations may be too great for geneflow to occur. In contrast, there was a lack of genetic partitioning for *A. mccullochi* populations. The difference between species in the level of gene flow between populations probably represents different geographic distances between the sample populations (McCulloch populations were 160 km apart and the akindynos sample populations were 1200 km apart). This gene flow between populations is promising for *A. mccullochi* since it facilitates recolonization if one population was to go locally extinct. However, only two locations in each species were sampled and further samples of *A. akindynos* from the Great Barrier Reef, southern reefs (e.g., Solitary Islands), and New Caledonia, as well as samples of *A. mccullochi* from Elizabeth Reef will be needed to fully quantify gene flow between all populations of these species.

### Genetic diversity

In both species, mtDNA data showed high genetic variability, *h* almost double and π close to an order of magnitude greater than the cut-off defined by Grant and Bowen (1998; both *h* and π >0.5). Similarly, high levels of genetic diversity for msatDNA were also found in both species. This suggests that populations of both species are either large and stable with long evolutionary histories, or that there has been secondary contact between differentiated lineages (Grant and Bowen 1998). Given that *A. mccullochi* does not have a large population (Choat et al. 2006; Hobbs and Feary 2007), secondary contact between differentiated lineages is more likely and is consistent with reticulate evolution between these sister species. Given that mtDNA diversity tracks with nuclear genetic diversity in many marine species (reviewed by Johannesson and Andre 2006), such high genetic diversity in *A. mccullochi* and *A. akindynos* is encouraging as it suggests that both species may have a greater adaptive capacity to deal with environmental change than if they had low genetic diversity. However, a cautious approach is still warranted given that quantitative trait loci under selection, can have no genetic diversity in peripheral populations despite high neutral genetic diversity (Kellermann et al. 2009).

Conserving genetic diversity is considered a priority by the IUCN (McNeely et al. 1990), and even more so for *A. mccullochi* given its vulnerability to extinction due to other traits (low abundance, small geographic range, and ecological specialization). The high genetic diversity of *A. mccullochi* appears to have arisen through complex reticulate evolution involving historical (but not contemporary) hybridization. High genetic diversity and population connectivity may reduce extinction risk in *A. mccullochi*, but identifying and protecting populations implicated in generating the complex reticulate structure among this species should be a conservation priority. Conserving *A. mccullochi* would best be achieved by protecting each of its MUs and by minimizing threats to population size, such as habitat loss (e.g., anemone bleaching) and collection for the aquarium trade.
